# Dietary patterns and the risk of tuberculosis-drug-induced liver injury: a cohort study

**DOI:** 10.3389/fnut.2024.1393523

**Published:** 2024-06-20

**Authors:** Jinyu Wang, Yarui Zhou, Cong Zhao, Ke Xiong, Yufeng Liu, Shanliang Zhao, Aiguo Ma

**Affiliations:** ^1^School of Public Health, Institute of Nutrition and Health, Qingdao University, Qingdao, China; ^2^Qingdao Chest Hospital, Qingdao, China; ^3^Linyi People’s Hospital, Linyi, China

**Keywords:** dietary pattern, liver injury, liver dysfunction, tuberculosis, cohort study

## Abstract

**Background and purpose:**

Nutrition is associated with tuberculosis drug-induced liver injury (TBLI). How dietary patterns relate to tuberculosis drug-induced liver injury is still unknown. The objective of this study is to explore the relation between dietary patterns and the risk of tuberculosis drug-induced liver injury.

**Methods:**

This cohort study was conducted at two hospitals in Shandong Province, China, between 2011 and 2013. A total of 605 tuberculosis patients were included in the final analysis. The blood aspartate aminotransferase or alanine aminotransferase level was monitored through the 6-month tuberculosis treatment. The semi-quantitative food frequency questionnaires were used to survey dietary intake in the second month of the tuberculosis treatment. The China Healthy Diet Index (CHDI), which was previously validated in the Chinese population, was used as an *a priori* dietary pattern. *A posteriori* dietary patterns were extracted by principal component analysis (PCA).

**Results:**

The CHDI was negatively associated with the risk of liver injury [adjusted odds ratio (aOR) per standard deviation (SD) (95% CI): 0.61 (0.40–0.94)] and liver dysfunction [aOR per SD (95% CI): 0.47 (0.35–0.64)] in the multivariate logistic model. A positive association between “Organ meat, poultry, and vegetable oil” dietary pattern scores (extracted by PCA) and the risk of liver injury [aOR (95% CI): 3.02 (1.42–6.41)] and liver dysfunction [aOR (95% CI): 1.83 (1.09–3.05)] was observed.

**Conclusion:**

In conclusion, a high CHDI score was a protective factor for tuberculosis drug-induced liver injury, while the “Organ meat, poultry, and vegetable oil” dietary pattern, which was rich in organ meat, poultry, and vegetable oil and low in vegetables, was an independent risk factor for tuberculosis drug-induced liver injury.

## 1 Introduction

Tuberculosis treatment is a leading cause of drug-induced liver injury (1). The major tuberculosis drugs include ethambutol (EMB), pyrazinamide (PZA), rifampicin (RIF), and isoniazid (INH). INH, RIF, and PZA are hepatotoxic, and the combination of these drugs can further exacerbate hepatotoxicity (2). During tuberculosis treatment, 5.0–33.0% of patients experienced liver injury (3). Clinical symptoms of tuberculosis drug-induced liver injury (TBLI) include jaundice, nausea, vomiting, rash, and pruritus (1). The liver injury results in an interruption of tuberculosis treatment, hindering the treatment effect, increasing the risk of drug resistance, and in severe cases, leading to acute liver failure and even death (4, 5).

No effective treatment exists for TBLI. Nutrition is closely related to TBLI (6). Epidemiological studies indicated that body mass index (BMI) was negatively associated with the risk of TBLI (7). Randomized controlled trials indicated that carnitine, jujube syrup, and *Lactobacillus casei* could provide protection against TBLI (8–10). Carnitine and jujube syrup alleviated hepatocellular damage-type TBLI, which manifested as significant elevations of alanine aminotransferase (ALT) and aspartate aminotransferase (AST) (8, 9). The *L. casei* intervention alleviated cholestasis-type TBLI (manifested as significant elevations of alkaline phosphatase and total bilirubin) by modulating the gut microbiota, reducing the blood liposaccharide content, and improving intestinal permeability (10). Animal studies reported that folic acid, vitamin B12, vitamin C, quercetin, hesperidin, curcumin, and beta-carotene could provide protection against TBLI (11–18). Nutritional interventions may be promising for mitigating TBLI (19) and other chronic diseases (20, 21).

Dietary pattern offers a holistic view of food consumption and overcomes the limitation of studying a single nutrient or food, which ignores the interactions among nutrients or foods (22, 23). Previous studies indicated that following a Mediterranean diet might lower the risk of non-alcoholic fatty liver disease (NAFLD), liver cancer, and liver fibrosis (24–26). Additionally, the healthy eating index (HEI) was negatively associated with the risk of hepatocellular carcinoma and NAFLD (27–31). The Western diet increased the risk of NAFLD, liver cirrhosis, and liver cancer, while the prudent diet reduced the risk of NAFLD, liver fibrosis, and cirrhosis (32–35). However, the relation between dietary patterns and TBLI was rarely reported.

The objective of the study is to investigate the associations of both *a priori*- and *a posteriori*-derived dietary patterns with TBLI. The China Healthy Diet Index (CHDI) was used in the study of a prior dietary pattern, which was developed based on the Dietary Guidelines for Chinese Residents (2016) in reference to the HEI-2010 (36). The CHDI was able to differentiate the diet quality of 55,528 participants from the Chinese nutrition and health surveillance (2010–2012) and was associated with a decreased risk of tuberculosis and hypertension (36, 37). Principal component analysis (PCA) was used to extract *a posteriori-*derived dietary pattern, which was commonly used in the nutrition literature (38, 39).

## 2 Materials and methods

### 2.1 Ethics

The study was approved by the Ethic Committee of Qingdao Center for Disease Control and Prevention (No. 2009-4). The study was conducted in accordance with the Declaration of Helsinki, and all participants provided informed consent. The trial was registered with the Chinese Clinical Trial Registry with the number ChiCTR-OCC-10000994.

### 2.2 Study design and population

The study was conducted at two hospitals in Shandong Province, China between 2011 and 2013. The inclusion criteria included being more than 18 years old and being newly diagnosed with pulmonary tuberculosis based on clinical symptoms, sputum smears, and computed tomography scans according to the “Technical Guidelines for Tuberculosis Prevention and Control in China” (40). The exclusion criteria included drug-resistant tuberculosis; concurrent liver, gastrointestinal, cardiovascular, respiratory diseases, cancer, human immunodeficiency virus (HIV), or mental disorders; pregnancy or nursing; AST and ALT levels above 40 U/L; and use of nutritional supplements during the past 2 months.

### 2.3 Procedure

The patients’ height and weight were measured upon admission to the hospital. The demographic information, which included sex, age, previous history of liver disease, diabetes status, education level, and outdoor exercise, was collected using a standard questionnaire. To assess participants’ dietary intake, a semi-quantitative food frequency questionnaire (FFQ) was conducted at the end of the second month of the tuberculosis treatment. The FFQ was previously validated (41). The FFQ included white flour, white rice, millet, corn, bran, dark vegetables, light vegetables, fruit, beef, pork, organ meat, lamb, tofu, beans, soybean milk, vegetable oil, animal oil, eggs, chicken, duck, fish, shrimp, potatoes, sweet potatoes, taro, yam, tea, dairy products, liquor, and beer. The frequency and amount of food consumption were surveyed. Food consumption was estimated in the unit of Liang (equivalent to 50 g). The consumption of tea and beer was estimated by cups. The food items were classified into 17 food groups, which included whole cereals, refined cereals, vegetables, fruit, red meat, organ meat, legumes, vegetable oil, animal oil, tea, fish and other seafood, eggs, tubers, liquor, beer, dairy products, and poultry according to the Dietary Guidelines for Chinese Residents (2016).

All patients received a standard tuberculosis treatment, which consisted of a 2-month intensive phase using EMB, PZA, RIF, and INH and a 4-month continuation phase with RIF and INH. The hospital personnel routinely tested the ALT, AST, and albumin (ALB) levels at 0, 1, 2, and 6 months after commencing the medication. An ALT or AST level greater than twice the upper limit of normal (ULN) indicated liver injury (42), while an ALT or AST level above the ULN indicated liver dysfunction. The ULNs of ALT and AST are 40 U/L (43).

### 2.4 Statistical analysis

The PCA with varimax rotation was used to extract the population-specific dietary patterns from 17 food groups. The analysis was tested using the Bartlett test of sphericity (*p* < 0.0001) and the Kaiser–Mayer–Olkin test (0.64, Supplementary Table S1). The dietary patterns with an eigenvalue ≥1.5 were extracted. The factor loadings reflect the magnitude of the relation between food groups and dietary patterns. The adherence score for each dietary pattern was calculated by the intake of each food group and the corresponding factor loadings. The CHDI was previously validated (36). The food intake was converted to per 1,000 kcal for scoring. The CHDI contains 13 items, including food variety, whole grain, refined grain, total vegetables, dark green and orange vegetables, dry bean and tuber, soybean, fruit, fish, dairy, meat and egg, shellfish and mollusk, sodium and empty calories, and calories from solid fats (SoFAS). The CHDI score ranges from 0 to 100. A higher score indicated a higher diet quality.

Statistical Package for the Social Sciences (SPSS) 26.0 was used for the statistical analysis. Inter-group differences were tested by a χ^2^ test (for proportions) or an ANOVA test (for continuous variables). The correlation was analyzed by Spearman’s correlation. A logistic regression analysis was used to explore the relationship between dietary patterns and the risk of liver injury and dysfunction. For each dietary pattern, the lowest tertile was used as the reference group. Age, sex, area, BMI, energy intake, and diabetes were adjusted as covariates. A *p*-value of <0.05 was considered indicative of statistical significance.

## 3 Results

### 3.1 *A priori and a posteriori* dietary patterns by CHDI and PCA

A total of 706 tuberculosis patients were recruited into the study (flow chart shown in Supplementary Figure S1). Among them, participants were excluded from the analysis due to no or incomplete FFQ (*n* = 38), extreme energy intake, no liver function results (*n* = 13), or changing treatment plan (*n* = 50). During the tuberculosis treatment of the participants, 49 cases of liver injury and 141 cases of liver dysfunction were identified.

Three main dietary patterns with an eigenvalue above 1.5 were extracted by PCA (Supplementary Figure S2). These three dietary patterns accounted for 34.62% of the total variations in food intake (Table 1). The first dietary pattern, labeled “Vegetables, red meat, fish, and other seafood,” was characterized by a high intake of vegetables, red meat, fish, other seafood, tubers, and tea and a low intake of liquor, refined cereals, and animal oil. The second dietary pattern, named “Organ meat, poultry, and vegetable oil,” was characterized by a high intake of organ meat, poultry, vegetable oil, and whole cereals and a low intake of animal oil and liquor. The third dietary pattern, labeled “Fruit, legumes, and eggs,” was characterized by a high intake of fruit, legumes, eggs, liquor, dairy products, and refined cereals.

**Table 1:** Characteristics of *a priori*- and *a posteriori*-derived dietary pattern identified among tuberculosis patients.

Food groups	Mean intake (g/d or ml/d)	China healthy diet index^a^	PCA^b,c^		
			“Vegetables, red meat, fish, and other seafood”	“Organ meat, poultry, and vegetable oil”	“Fruits, legumes, and eggs”
Vegetables	225.3	+	**0.75**	−0.19	0.06
Fruits	92.8	+	0.12	0.03	**0.61**
Legumes	18.9	+	0.08	0.28	**0.55**
Tubers	51.3	+	0.43	0.27	0.00
Eggs	49.8	+	0.25	0.09	**0.52**
Whole cereals	57.9	+	0.11	0.50	−0.06
Refined cereals	349.0	+	−0.35	0.14	0.41
Fish and other seafood	26.4	+	**0.56**	0.36	0.21
Red meat	73.7	+	**0.63**	0.02	0.27
Poultry	20.2	+	0.20	**0.60**	0.06
Organ meat	7.9	+	−0.10	**0.70**	0.16
Animal oil	10.5	−	−0.30	−0.26	0.28
Vegetable oil	30.3	−	−0.16	**0.55**	−0.04
Dairy products	61.2	+	0.29	0.05	0.45
Tea	0.5	Not included	0.33	−0.02	0.00
Liquor	63.4	−	−0.37	−0.24	0.50
Beer	7.8	−	−0.02	−0.10	0.23
	Eigen value		2.62	1.67	1.60
Explained variation in food group intake (%)			15.39	9.83	9.40

^a^Food groups have positive (+) or negative (−) contributions to the China healthy diet index (CHDI).

^b^The top three food groups (with the highest factor loading) are shown in bold.

^c^The dietary patterns were obtained by principal component analyses with Varimax rotation.

We performed the Spearman correlation analysis between the dietary pattern score and nutrient intakes (Table 2). The CHDI was positively correlated with vitamin C (*r* = 0.66), animal protein (*r* = 0.65), niacin (*r* = 0.58), vitamin A (*r* = 0.57), riboflavin (*r* = 0.55), Ca (*r* = 0.50), Zn (*r* = 0.49), cholesterol (*r* = 0.48), and K (*r* = 0.44). The “Vegetables, red meat, fish and other seafood” dietary pattern score was positively correlated with vitamin C (*r* = 0.77), animal protein (*r* = 0.66), vitamin A (*r* = 0.54), niacin (*r* = 0.50), cholesterol (*r* = 0.49), riboflavin (*r* = 0.42), Zn (*r* = 0.41), and Ca (*r* = 0.40) and negatively correlated with vegetable protein (*r* = −0.40). The “Organ meat, poultry, and vegetable oil” dietary pattern score was positively correlated with fat (*r* = 0.49), vitamin E (*r* = 0.46), riboflavin (*r* = 0.42), and Fe (*r* = 0.41). The “Fruit, liquor, and legumes” dietary pattern score was positively correlated with Ca (*r* = 0.74), P (*r* = 0.70), Se (*r* = 0.70), riboflavin (*r* = 0.65), K (*r* = 0.66), energy (*r* = 0.62), Zn (*r* = 0.62), dietary fiber (*r* = 0.59), Cu (*r* = 0.59), animal protein (*r* = 0.58), cholesterol (*r* = 0.55), fat (*r* = 0.53), thiamin (*r* = 0.52), niacin (*r* = 0.49), Na (*r* = 0.44), Mg (*r* = 0.44), Fe (*r* = 0.44), Mn (*r* = 0.44), and carbohydrate (*r* = 0.40).

**Table 2:** The Spearman rank correlation coefficients between dietary pattern scores and nutrient intakes.

	China healthy diet index	“Vegetables, red meat, fish, and other seafood”	“Organ meat, poultry, and vegetable oil”	“Fruit, legumes, and eggs”
Vegetable protein	−0.23	**−0.40**	0.17	0.31
Animal protein	**0.65** ^a^	**0.66**	0.29	**0.58**
Energy	0.06	−0.10	0.29	**0.62**
Fat	0.25	0.20	**0.49**	**0.53**
Carbohydrate	−0.09	−0.31	0.08	**0.40**
Dietary fiber	0.37	0.24	0.29	**0.59**
Cholesterol	**0.48**	**0.49**	0.34	**0.55**
Vitamin A	**0.57**	**0.54**	0.39	0.37
Thiamin	0.32	0.19	0.21	**0.52**
Riboflavin	**0.55**	**0.42**	**0.42**	**0.65**
Niacin	**0.58**	**0.50**	0.32	**0.49**
Vitamin C	**0.66**	**0.77**	−0.05	0.18
Vitamin E	−0.22	−0.21	**0.46**	0.12
Ca	**0.50**	**0.40**	0.24	**0.74**
P	0.37	0.23	0.38	**0.70**
K	**0.44**	0.36	0.36	**0.66**
Na	0.31	0.29	0.26	**0.44**
Mg	0.08	−0.04	0.33	**0.54**
Fe	−0.09	−0.18	**0.41**	**0.44**
Zn	**0.49**	**0.41**	0.39	**0.62**
Se	0.36	0.24	0.37	**0.70**
Cu	0.17	0.05	0.39	**0.59**
Mn	0.00	−0.15	0.34	**0.44**

^a^Nutrient intakes with correlation coefficients (absolute values) ≥ 0.4 in each dietary pattern are shown in bold.

### 3.2 Baseline characteristics of the participants

The baseline characteristics of the participants are shown by tertiles of both *a priori* and *a posteriori* dietary pattern scores in Table 3. For the *a priori* dietary pattern, patients in the highest tertile of CHDI were younger, had a higher BMI, had more residents from the Qingdao area, and had a higher prevalence of diabetes than patients in the lowest tertile. For the *a posteriori* dietary pattern, patients in the highest tertile of “Vegetables, red meat, fish, and other seafood” dietary pattern score were younger, had a higher BMI, comprised more male individuals, had a higher prevalence of diabetes, and had more residents from the Qingdao area than patients in the lowest tertile. Patients in the highest tertile of “Organ meat, poultry, and vegetable oil” dietary pattern score had more residents from the Linyi area and a lower prevalence of diabetes. Patients in the highest tertile of “Fruit, legumes, and eggs” dietary pattern score had more residents from the Qingdao area and a higher prevalence of diabetes.

**Table 3:** Characteristics of participants (*n* = 605) based on the tertiles of dietary pattern scores.

Dietary pattern	Characteristic		Tertile 1 (lowest) % or mean ± SD	Tertile 2% or mean ± SD	Tertile 3 (highest) % or mean ± SD	*p* value^a,b^
China healthy diet index	Age		50.6 ± 19.4	46.4 ± 19.3	45.2 ± 18.2	0.010
	Male		73.3	72.3	72.6	0.975
	BMI		20.7 ± 2.8	20.2 ± 2.8	21.5 ± 3.1	<0.001
	Area	Qingdao	4.5	18.3	81.6	<0.001
		Linyi	95.5	81.7	18.4	
	Illiteracy		16.8	12.9	10.0	0.123
	Diabetes		4.0	5.9	40.0	<0.001
“Vegetables, red meat, fish, and other seafood”	Age		50.2 ± 19.1	47.5 ± 19.7	44.5 ± 18.0	0.010
	Male		75.7	64.7	77.7	0.007
	BMI		20.4 ± 2.8	20.7 ± 2.8	21.4 ± 3.2	<0.001
	Area	Qingdao	3.0	21.9	79.2	<0.001
		Linyi	97.0	78.1	20.8	
	Illiteracy		17.8	10.9	10.9	0.061
	Diabetes		5.0	8.0	36.6	<0.001
“Organ meat, poultry, and vegetable oil”	Age		47.8 ± 19.1	45.2 ± 19.2	49.2 ± 18.8	0.103
	Male		74.8	73.3	70.1	0.571
	BMI		21.2 ± 2.8	20.5 ± 3.0	20.8 ± 3.0	0.058
	Area	Qingdao	38.1	38.1	27.9	0.044
		Linyi	61.9	61.9	72.1	
	Illiteracy		15.3	10.9	13.4	0.415
	Diabetes		21.3	17.4	11.0	0.020
“Fruit, legumes, and eggs”	Age		47.7 ± 20.2	46.9 ± 19.0	47.6 ± 18.0	0.900
	Male		32.2	26.9	22.8	0.104
	BMI		20.5 ± 2.9	21.0 ± 3.0	20.9 ± 3.0	0.211
	Area	Qingdao	20.3	41.3	42.6	<0.001
		Linyi	79.7	58.7	57.4	
	Illiteracy		14.9	12.4	12.4	0.704
	Diabetes		7.0	17.4	25.2	<0.001

^a^*p* values were obtained by a χ^2^ test for proportions and an ANOVA test for continuous variables.

^b^Statistically significant values (*p* < 0.05) are shown in bold characters. SD, Standard deviation.

### 3.3 Associations between dietary patterns and TBLI

The associations between dietary patterns and the risk of liver injury and liver dysfunction are shown in Tables 4, 5, respectively. For the *a priori* dietary pattern, the CHDI was negatively associated with the risk of liver injury [adjusted odds ratio (aOR) per SD (95% CI): 0.61 (0.40–0.94)] and liver dysfunction [aOR per SD (95% CI): 0.47 (0.35–0.64)].

**Table 4:** Risk of liver injury according to the dietary pattern scores.^a,b^

		Tertiles		*p* value for trend	OR per SD	*p* value
	Tertile 1 (lowest)	Tertile 2	Tertile 3 (highest)			
China healthy diet index						
Liver injury/non-liver injury	11/191	18/184	20/181			
Crude model	1.0	1.70 (0.78–3.69)	1.92 (0.89–4.12)	0.119	1.24 (0.92–1.65)	0.156
Model 1	1.0	1.08 (0.46–2.49)	0.42 (0.15–1.16)	0.043	0.63 (0.42–0.94)	0.024
Model 2	1.0	1.15 (0.49–2.68)	0.40 (0.14–1.12)	0.037	0.61 (0.40–0.94)	0.023
“Vegetables, red meat, fish, and other seafood”						
Liver injury/non-liver injury	4/198	20/181	25/177			
Crude model	1.0	5.47 (1.84–16.31)	6.99 (2.39–20.48)	0.001	1.41 (1.08–1.84)	0.012
Model 1	1.0	4.34 (1.41–13.35)	3.02 (0.87–10.56)	0.532	0.90 (0.62–1.31)	0.582
Model 2	1.0	4.52 (1.40–14.60)	3.05 (0.86–10.82)	0.526	0.91 (0.61–1.36)	0.658
“Organ meat, poultry, and vegetable oil”						
Liver injury/non-liver injury	13/189	11/191	25/176			
Crude model	1.0	0.84 (0.37–1.92)	2.07 (1.03–4.16)	0.012	1.21 (0.96–1.54)	0.108
Model 1	1.0	0.80 (0.35–1.87)	2.51 (1.22–5.16)	0.002	1.42 (1.10–1.82)	0.007
Model 2	1.0	0.86 (0.37–2.00)	3.02 (1.42–6.41)	0.001	1.65 (1.21–2.24)	0.002
“Fruit, legumes, and eggs”						
Liver injury/non-liver injury	16/186	16/185	17/185			
Crude model	1.0	1.01 (0.49–2.07)	1.07 (0.52–2.18)	0.846	0.98 (0.72–1.32)	0.877
Model 1	1.0	0.71 (0.34–1.52)	0.75 (0.35–1.58)	0.550	0.88 (0.60–1.30)	0.524
Model 2	1.0	0.70 (0.32–1.53)	0.78 (0.33–1.85)	0.692	0.90 (0.54–1.50)	0.686

^a^Aspartate aminotransferase (AST) or alanine aminotransferase (ALT) levels greater than twice the upper limit of normal (ULN) indicate liver injury. The ULN for ALT and AST is 40 U/L.

^b^Model 1 was adjusted for age, gender, and area. Model 2 was additionally adjusted for BMI, energy intake, and diabetes status.

**Table 5:** Risk of liver dysfunction according to the dietary pattern scores.^a,b^

		Tertiles		*p* value for trend	OR per SD	*p* value
	Tertile 1 (lowest)	Tertile 2	Tertile 3 (highest)			
China healthy diet index						
Liver dysfunction/non-liver dysfunction	43/159	44/158	54/147			
Crude model	1.0	1.03 (0.64–1.66)	1.36 (0.86–2.15)	0.162	1.14 (0.94–1.37)	0.180
Model 1	1.0	0.62 (0.36–1.07)	0.20 (0.09–0.42)	<0.001	0.53 (0.40–0.70)	<0.001
Model 2	1.0	0.56 (0.32–0.97)	0.15 (0.07–0.33)	<0.001	0.47 (0.35–0.64)	<0.001
“Vegetables, red meat, fish, and other seafood”						
Liver dysfunction/non-liver dysfunction	31/171	47/154	63/139			
Crude model	1.0	1.68 (1.02–2.78)	2.50 (1.54–4.06)	<0.001	1.30 (1.08–1.57)	0.005
Model 1	1.0	1.23 (0.71–2.11)	0.76 (0.39–1.50)	0.813	0.99 (0.95–1.00)	0.145
Model 2	1.0	1.50 (0.84–2.67)	0.85 (0.43–1.71)	0.327	0.80 (0.62–1.04)	0.092
“Organ meat, poultry, and vegetable oil”						
Liver dysfunction/non-liver dysfunction	39/163	46/156	56/145			
Crude model	1.0	1.23 (0.76–1.99)	1.61 (1.01–2.57)	0.044	1.15 (0.97–1.38)	0.110
Model 1	1.0	1.22 (0.74–2.02)	1.98 (1.21–3.24)	0.005	0.99 (0.98–1.00)	0.116
Model 2	1.0	1.25 (0.75–2.07)	1.83 (1.09–3.05)	0.020	1.28 (1.04–1.57)	0.020
“Fruit, legumes, and eggs”						
Liver dysfunction/non-liver dysfunction	4/162	53/148	48/154			
Crude model	1.0	1.45 (0.91–2.31)	1.26 (0.79–2.03)	0.472	1.12 (0.94–1.33)	0.218
Model 1	1.0	1.08 (0.66–1.77)	0.92 (0.55–1.51)	0.623	0.99 (0.98–1.00)	0.154
Model 2	1.0	0.93 (0.56–1.55)	0.67 (0.37–1.20)	0.147	0.87 (0.66–1.15)	0.329

^a^Aspartate aminotransferase (AST) or alanine aminotransferase (ALT) levels above upper limit of normal (ULN) indicated liver dysfunction. The ULN for ALT and AST is 40 U/L.

^b^Model 1 was adjusted for age, gender, and area. Model 2 was additionally adjusted for BMI, energy intake, and diabetes status.

For the *a posteriori* dietary pattern, the patients in the highest tertile of the “Organ meat, poultry, and vegetable oil” dietary pattern score had a higher risk of liver injury [aOR (95% CI): 3.02 (1.42–6.41)] and liver dysfunction [aOR (95% CI): 1.83 (1.09–3.05)] than patients in the lowest tertile after adjusting for age, gender, area, BMI, energy intake, and diabetes status. During tuberculosis treatment, the “Vegetables, red meat, fish, and other seafood” and “Fruit, legumes, and eggs” dietary patterns were not associated with liver injury/dysfunction after adjustment for confounders.

We conducted subgroup analyses to assess whether the associations between dietary pattern scores and the risk of TBLI/tuberculosis drug-induced liver dysfunction varied by age, sex, area, BMI, diabetes, and smoking status (Supplementary Tables S2, S3). After adjusting for covariates, the negative association between the CHDI score and the risk of TBLI/tuberculosis drug-induced liver dysfunction was particularly significant in male patients, Qingdao residents, patients with a BMI of <24, and patients without diabetes or smoking. The positive association between the “Organ meat, poultry, and vegetable oil” pattern and the risk of TBLI/tuberculosis drug-induced liver dysfunction was particularly significant in male patients, younger patients (≤ 65 years), Linyi residents, patients with a BMI of <24, and patients without diabetes.

## 4 Discussion

To our knowledge, this study is one of the first to investigate the relation between dietary patterns and the risk of TBLI. Our results suggested that the *a priori* dietary pattern, based on the CHDI, was negatively associated with the risk of TBLI, while the “Organ meat, poultry, and vegetable oil” dietary pattern, extracted by PCA, was positively associated with the risk of TBLI.

Our results showed a negative relation between the CHDI and the risk of TBLI. The CHDI is developed in reference to the HEI-2010, both of which grant high scores for the high intake of whole grain, total vegetables, dark vegetables, fruit, dairy products, meat, eggs, fish, and other seafood and for the low intake of animal oil and sodium (36). Our results indicated that a balanced diet (e.g., a diet with a high CHDI score) may be beneficial for alleviating TBLI. Consistently, previous studies reported that another balanced diet, a diet with a high HEI score, was negatively associated with other liver diseases such as NAFLD and hepatocellular carcinoma (29, 31). A cohort study including 4,94,942 participants found that adherence to HEI may reduce the risk of developing hepatocellular carcinoma and the risk of dying from chronic liver disease (29). A cross-sectional study including 2,892 participants found that HEI was associated with a reduced incidence of NAFLD (31).

For the *posteriori* dietary patterns extracted by PCA, our results suggested that the “Organ meat, poultry, and vegetable oil” dietary pattern was positively associated with the risk of TBLI and tuberculosis drug-induced liver dysfunction. The associations became stronger after adjusting for confounders including age, gender, area, BMI, energy intake, and diabetes status. Subgroup analyses indicated that the positive associations were observed between the “Organ meat, poultry, and vegetable oil” dietary pattern and the risk of TBLI and tuberculosis drug-induced liver dysfunction were particularly significant in male patients, Linyi residents, and participants aged ≤65 years. In addition, strongly positive associations between the “Organ meat, poultry, and vegetable oil” dietary pattern and the dietary intake of total fat, riboflavin, vitamin E, and iron were observed. Consistently, the intake of vegetable oil was negatively correlated with TBLI, as indicated in our previous research (44).

Several mechanisms may explain the observed associations between dietary patterns and TBLI in our study. First, diseases such as TBLI are closely related to oxidative stress and chronic inflammation (19, 45). A higher diet quality score (such as those rated by a higher HEI or CHDI score) was associated with a reduced level of oxidative stress and inflammation biomarkers (46, 47). Thus, a diet with a higher CHDI score may alleviate TBLI by reducing oxidative stress and inflammation. Second, the major individual foods comprising the dietary patterns may contribute to the associations with TBLI. Vegetable intake was associated with a reduced risk of TBLI in our previous cohort study, which may be attributed to the phytochemicals in vegetables (44). *In vitro* and animal studies showed that phytochemicals could reduce free radicals and inflammation (48). On the other hand, organ meat and vegetable oil consumption were positively associated with NAFLD and TBLI, respectively (44, 49). Vegetable oil is enriched with linoleic acid, which may induce liver injury by increasing the activity of cytochrome P450 2E1 and inducing liver inflammation (50). Thus, the “Organ meat, poultry, and vegetable oil” dietary pattern, which is characterized by a high intake of organ meat, poultry, and vegetable oil, was associated with an increased risk of TBLI.

The major strengths of the current study are the following. First, we investigated the associations of both *a priori* and *posteriori* dietary patterns with TBLI, which represent a comprehensive approach. Second, in the investigation of the *a priori* dietary pattern, a previously validated and specifically designed index for the Chinese population, the CHDI, was used (36). Third, detailed demographic information was systematically collected during the study, which allowed us to adjust for common confounding variables associated with TBLI, including BMI, sex, age, location, energy intake, and diabetes.

The limitations should be acknowledged. First, the generalizability of the study needs future study because all the included subjects were Chinese. Second, due to the low incidence of tuberculosis (58/1,000,000 in China), the sample size was relatively small (51), which may weaken the statistical power. Third, the study was observational, and no causal relationship could be drawn. However, we carefully adjusted for common confounding factors.

In conclusion, a higher CHDI score was associated with a reduced risk of TBLI, while the “Organ meat, poultry, vegetable oil” dietary pattern, which was rich in organ meat, poultry, and vegetable oil and low in vegetables, was positively associated with the risk of TBLI. A diet with a high CHDI score and involving less organ meat and vegetable oil may be recommended during tuberculosis treatment to prevent TBLI. Future studies may validate this conclusion in a non-Chinese population with a larger sample size.

## Data availability statement

The raw data supporting the conclusions of this article will be made available by the authors, without undue reservation.

## Ethics statement

The studies involving humans were approved by the Ethic Committee of Qingdao Center for Disease Control and Prevention. The studies were conducted in accordance with the local legislation and institutional requirements. The participants provided their written informed consent to participate in this study.

## Author contributions

JW: Funding acquisition, Writing – original draft, Data curation, Formal analysis. YZ: Writing – original draft, Data curation, Formal analysis. CZ: Investigation, Writing – review & editing. KX: Writing – review & editing. YL: Investigation, Writing – review & editing. SZ: Investigation, Writing – review & editing. AM: Writing – review & editing, Funding acquisition, Conceptualization.
