# Supplementary material for: Dietary patterns and the risk of tuberculosis-drug-induced liver injury: a cohort study
Source: Front Nutr. 2024 Jun 20;11:1393523. doi: 10.3389/fnut.2024.1393523 (PMC11223592; doi:10.3389/fnut.2024.1393523)
Supplement: Supplementary file 1 [file Data_Sheet_1.docx]

Supplementary Material

# Table S1. Factorability of the correlation matrix of the original variables: individual and overall measures of sampling adequacy^a^ and Bartlett’s test of sphericity.

| Bartlett’s test of sphericity: *P*-value < 0.0001 | | |
| --- | --- | --- |
| Kaiser–Meyer–Olkin statistic—overall measure of sampling adequacy^a^: 0.64 | | |
| Individual measures of sampling adequacy^a^: | | |
|  | Mediocre: <0.20 | Beer, tea |
|  | Mediocre: 0.20-0.29 | Dairy products, animal oil, tubers, whole cereals |
|  | Mediocre: 0.30-0.39 | Eggs, fruit, legumes, vegetable oil, refined cereals |
|  | Mediocre: 0.40-0.49 | Red meats, poultry, fish and other seafood, liquor |
|  | Mediocre: 0.50-0.59 | Organ meats |
|  | Mediocre:>0.6 | Vegetables |

a. Overall and individual measures of sampling adequacy range between 0 and 1.

# Table S2. Multivariate odds ratio of liver injury stratified by age, gender, area, BMI, diabetes, smoking.

|  |  | **Multivariate relative risk (95% CI) per tertiles^a^** | | | ***P* values for trend** |  | **OR per SD** | ***P* values for interaction** |
| --- | --- | --- | --- | --- | --- | --- | --- | --- |
|  |  | **Tertile 1 (lowest)** | **Tertile 2** | **Tertile 3 (highest)** |  |  |  |  |
| **China healthy diet index** |  |  |  |  |  |  |  |  |
| Age (years) |  |  |  |  |  |  |  |  |
| ≤65 | Crude model | 1.0 | 1.27 (0.56-2.86) | 1.66 (0.77-3.59) | 0.190 |  | 1.19 (0.86-1.62) | 0.975 |
|  | Model 1 | 1.0 | 0.88 (0.37-2.11) | 0.47 (0.17-1.32) | 0.115 |  | 0.66 (0.43-1.00) | 0.999 |
|  | Model 2 | 1.0 | 0.95 (0.39-2.27) | 0.47 (0.17-1.34) | 0.116 |  | 0.66 (0.42-1.02) | 0.991 |
| >65 | Crude model | 1.0 | - | - | 0.984 |  | 1.09 (0.39-3.07) |  |
|  | Model 1 | 1.0 | - | - | 0.050 |  | 0.23 (0.05-1.12) |  |
|  | Model 2 | 1.0 | - | - | 0.075 |  | 0.15 (0.02-1.41) |  |
| Gender |  |  |  |  |  |  |  |  |
| Male | Crude model | 1.0 | 2.31 (0.91-5.84) | 2.48 (0.99-6.22) | 0.081 |  | 1.27 (0.92-1.75) | 0.347 |
|  | Model 1 | 1.0 | 1.17 (0.41-3.33) | 0.43 (0.12-1.50) | 0.058 |  | **0.58 (0.36-0.91)** | 0.570 |
|  | Model 2 | 1.0 | 1.24 (0.43-3.55) | 0.41 (0.12-1.48) | 0.055 |  | **0.57 (0.35-0.92)** | 0.539 |
| Female | Crude model | 1.0 | 0.71 (0.15-3.32) | 0.98 (0.23-4.14) | 0.964 |  | 1.14 (0.59-2.18) |  |
|  | Model 1 | 1.0 | 0.71 (0.15-3.37) | 0.45 (0.07-2.90) | 0.150 |  | 0.80 (0.34-1.86) |  |
|  | Model 2 | 1.0 | 0.82 (0.16-4.18) | 0.44 (0.06-3.27) | 0.424 |  | 0.81 (0.31-2.07) |  |
| Area |  |  |  |  |  |  |  |  |
| Qingdao | Crude model | 1.0 | 1.13 (0.20-6.42) | 0.49 (0.09-2.51) | 0.064 |  | **0.53 (0.32-0.87)** | 0.571 |
|  | Model 1 | 1.0 | 0.90 (0.15-5.48) | 0.43 (0.08-2.30) | 0.074 |  | **0.34 (0.33-0.89)** | 0.610 |
|  | Model 2 | 1.0 | 1.09 (0.17-6.98) | 0.43 (0.08-2.44) | 0.056 |  | **0.52 (0.30-0.89)** | **0.027** |
| Linyi | Crude model | 1.0 | 1.18 (0.46-3.04) | 0.00 (0.00--) | 0.365 |  | 0.89 (0.46-1.73) |  |
|  | Model 1 | 1.0 | 1.16 (0.45-3.02) | 0.00 (0.00--) | 0.350 |  | 0.87 (0.44-1.72) |  |
|  | Model 2 | 1.0 | 1.24 (0.47-3.31) | 0.00 (0.00--) | 0.376 |  | 0.86 (0.42-1.76) |  |
| BMI (kg/m^2^) |  |  |  |  |  |  |  |  |
| <24 | Crude model | 1.0 | 1.98 (0.82-4.79) | 2.10 (0.86-5.15) | 0.139 |  | 1.26 (0.90-1.75) | 0.424 |
|  | Model 1 | 1.0 | 1.21 (0.47-3.16) | 0.41 (0.13-1.31) | 0.058 |  | **0.59 (0.38-0.93)** | 0.488 |
|  | Model 2 | 1.0 | 1.21 (0.46-3.15) | 0.39 (0.12-1.28) | 0.056 |  | **0.58 (0.36-0.92)** | 0.488 |
| ≥24 | Crude model | 1.0 | 0.90 (0.16-5.04) | 0.97 (0.22-4.34) | 0.997 |  | 0.97 (0.51-1.86) |  |
|  | Model 1 | 1.0 | 0.66 (0.10-4.29) | 0.44 (0.05-3.71) | 0.459 |  | 0.71 (0.29-1.72) |  |
|  | Model 2 | 1.0 | 0.90 (0.12-6.51) | 0.62 (0.06-5.95) | 0.646 |  | 0.82 (0.31-2.17) |  |
| Diabetes |  |  |  |  |  |  |  |  |
| No | Crude model | 1.0 | 1.52 (0.69-3.37) | 1.34 (0.54-3.34) | 0.555 |  | 1.16 (0.80-1.66) | 0.605 |
|  | Model 1 | 1.0 | 1.02 (0.43-2.41) | **0.31 (0.10-0.99)** | **0.035** |  | **0.62 (0.39-0.99)** | 0.400 |
|  | Model 2 | 1.0 | 1.11 (0.47-2.64) | **0.29 (0.09-0.94)** | **0.027** |  | **0.60 (0.37-0.97)** | 0.350 |
| Yes | Crude model | 1.0 | - | - | 0.485 |  | 1.00 (0.51-1.96) |  |
|  | Model 1 | 1.0 | - | - | 0.584 |  | 0.60(0.25-1.44) |  |
|  | Model 2 | 1.0 | - | - | 0.710 |  | 0.62 (0.23-1.65) |  |
| Smoking |  |  |  |  |  |  |  |  |
| No | Crude model | 1.0 | 1.37 (0.55-3.41) | 1.65 (0.69-3.91) | 0.269 |  | 1.17 (0.84-1.63) | 0.371 |
|  | Model 1 | 1.0 | 0.82 (0.30-2.22) | 0.34 (0.11-1.04) | **0.030** |  | **0.59 (0.38-0.91)** | 0.220 |
|  | Model 2 | 1.0 | 0.88 (0.32-2.41) | 0.33 (0.11-1.04) | **0.027** |  | **0.57 (0.36-0.90)** | 0.209 |
| Yes | Crude model | 1.0 | 2.98 (0.68-13.17) | 3.49 (0.53-22.97) | 0.148 |  | 1.71 (0.82-3.55) |  |
|  | Model 1 | 1.0 | 2.07 (0.41-10.40) | 0.76 (0.05-10.95) | 0.916 |  | 0.87 (0.30-2.53) |  |
|  | Model 2 | 1.0 | 2.34 (0.44-12.39) | 0.63 (0.03-13.01) | 0.927 |  | 1.01 (0.32-3.24) |  |
| **“Vegetables, red meat, fish, and other seafood”** |  |  |  |  |  |  |  |  |
| Age (years) |  |  |  |  |  |  |  |  |
| ≤65 | Crude model | 1.0 | **4.90 (1.62-14.84)** | **5.92 (2.01-17.48)** | **0.003** |  | **1.38 (1.04-1.83)** | 0.826 |
|  | Model 1^b^ | 1.0 | **4.13 (1.32-12.88)** | 3.06 (0.87-10.76) | 0.431 |  | 0.94 (0.64-1.39) | 0.827 |
|  | Model 2 | 1.0 | **4.08 (1.23-13.48)** | 3.07 (0.86-10.95) | 0.412 |  | 0.94 (0.61-1.45) | 0.847 |
| >65 | Crude model | 1.0 | -^c^ | - | 0.377 |  | 1.22 (0.40-3.69) |  |
|  | Model 1 | 1.0 | - | - | 0.522 |  | 0.39 (0.08-1.98) |  |
|  | Model 2 | 1.0 | - | - | 0.575 |  | 0.75 (0.27-2.10) |  |
| Gender |  |  |  |  |  |  |  |  |
| Male | Crude model | 1.0 | **6.03 (1.69-21.49)** | **7.72 (2.25-26.46)** | **0.002** |  | **1.46 (1.09-1.95)** | 0.657 |
|  | Model 1 | 1.0 | **4.32 (1.16-16.04)** | 2.85 (0.68-12.06) | 0.692 |  | 0.91 (0.60-1.38) | 0.673 |
|  | Model 2 | 1.0 | **4.74 (1.19-18.85)** | 2.98 (0.69 (12.91) | 0.684 |  | 0.94 (0.61-1.45) | 0.644 |
| Female | Crude model | 1.0 | 4.43 (0.52-38.02) | 4.68 (0.50-43.58) | 0.264 |  | 1.12 (0.56-2.27) |  |
|  | Model 1 | 1.0 | 4.09 (0.46-36.22) | 3.47 (0.26-46.04) | 0.577 |  | 0.77 (0.29-2.08) |  |
|  | Model 2 | 1.0 | 3.64 (0.39-33.68) | 3.45 (0.26-45.98) | 0.525 |  | 0.75 (0.27-2.10) |  |
| Area |  |  |  |  |  |  |  |  |
| Qingdao | Crude model | 1.0 | 1.47 (0.15-14.09) | 0.71 (0.08-6.43) | 0.121 |  | 0.64 (0.40-1.04) | **0.001** |
|  | Model 1 | 1.0 | 1.47 (0.15-14.50) | 0.66 (0.07-6.07) | 0.087 |  | 0.63 (0.38-1.03) | **0.002** |
|  | Model 2 | 1.0 | 1.49 (0.15-15.13) | 0.70 (0.07-6.93) | 0.165 |  | 0.62 (0.33-1.17) | **0.002** |
| Linyi | Crude model | 1.0 | **4.38 (1.18-16.19)** | **8.69 (1.99-37.96)** | **0.003** |  | **2.21 (1.06-4.58)** |  |
|  | Model 1 | 1.0 | **4.32 (1.16-16.07)** | **9.01 (2.04-39.69)** | **0.003** |  | **2.23 (1.07-4.66)** |  |
|  | Model 2 | 1.0 | **5.17(1.29-20.82)** | **10.20 (2.20-47.29)** | **0.002** |  | **2.30 (1.11-4.77)** |  |
| BMI (kg/m^2^) |  |  |  |  |  |  |  |  |
| <24 | Crude model | 1.0 | **6.30 (1.81-21.91)** | **7.05 (2.03-24.55)** | **0.005** |  | **1.43 (1.05-1.93)** | 0.952 |
|  | Model 1 | 1.0 | **6.32 (1.81-22.09)** | **6.32 (1.80-22.13)** | 0.972 |  | 0.88 (0.58-1.36) | 0.908 |
|  | Model 2 | 1.0 | **5.13 (1.33-19.73)** | 2.57 (0.60-11.03) | 0.967 |  | 0.88 (0.58-1.38) | 0.901 |
| ≥24 | Crude model | 1.0 | 2.86 (0.27-29.80) | 4.71 (0.55-40.44) | 0.139 |  | 1.16 (0.65-2.09) |  |
|  | Model 1 | 1.0 | 2.83 (0.27-30.10) | 4.81 (0.56-41.47) | 0.121 |  | 0.99 (0.42-2.35) |  |
|  | Model 2 | 1.0 | 2.69 (0.23-31.79) | 14.50 (0.62-340.74) | 0.091 |  | 1.17 (0.45-3.06) |  |
| Diabetes |  |  |  |  |  |  |  |  |
| No | Crude model | 1.0 | **4.76 (1.57-14.43)** | **6.21 (2.01-19.16)** | **0.003** |  | **1.42 (1.03-1.95)** | 0.551 |
|  | Model 1 | 1.0 | **3.97 (1.27-12.42)** | 3.17 (0.85-11.79) | 0.336 |  | 0.96 (0.62-1.48) | 0.714 |
|  | Model 2 | 1.0 | **4.11 (1.25-13.51)** | 3.20 (0.85-12.15) | 0.357 |  | 0.97 (0.61-1.54) | 0.753 |
| Yes | Crude model | 1.0 | - | - | 0.585 |  | 1.02 (0.53-1.97) |  |
|  | Model 1 | 1.0 | - | - | 0.462 |  | 0.62 (0.28-1.36) |  |
|  | Model 2 | 1.0 | - | - | 0.596 |  | 0.55 (0.19-1.55) |  |
| Smoking |  |  |  |  |  |  |  |  |
| No | Crude model | 1.0 | **3.71 (1.03-13.32)** | **5.77 (1.70-19.66)** | **0.004** |  | 1.36 (1.00-1.86) | 0.383 |
|  | Model 1 | 1.0 | 2.71 (0.72-10.14) | 2.17 (0.52-9.02) | 0.734 |  | 0.81 (0.52-1.27) | 0.213 |
|  | Model 2 | 1.0 | 2.65 (0.67-10.41) | 2.17 (0.51-9.19) | 0.703 |  | 0.83 (0.52-1.34) | 0.206 |
| Yes | Crude model | 1.0 | **14.60 (1.73-123.31)** | **13.27 (1.11-158.93)** | **0.027** |  | 1.77 (0.97-3.26) |  |
|  | Model 1 | 1.0 | **11.62 (1.31-103.53)** | 6.72 (0.42-106.55) | 0.300 |  | 1.24 (0.57-2.71) |  |
|  | Model 2 | 1.0 | **20.08 (1.48-272.85)** | 8.64 (0.45-166.09) | 0.237 |  | 1.32 (0.53-3.28) |  |
| **“Organ meat, poultry, and vegetable oil”** |  |  |  |  |  |  |  |  |
| Age (years) |  |  |  |  |  |  |  |  |
| ≤65 | Crude model | 1.0 | 0.74 (0.32-1.75) | 2.01 (0.98-4.14) | **0.015** |  | 1.22 (0.95-1.55) | 0.460 |
|  | Model 1 | 1.0 | 0.74 (0.31-1.76) | **2.40 (1.14-5.04)** | **0.004** |  | **1.38 (1.06-1.78)** | 0.478 |
|  | Model 2 | 1.0 | 0.79 (0.33-1.88) | **2.87 (1.33 -6.20)** | **0.001** |  | **1.59 (1.16-2.18)** | 0.446 |
| >65 | Crude model | 1.0 | - | - | 0.262 |  | 1.43 (0.57-3.59) |  |
|  | Model 1 | 1.0 | - | - | 0.220 |  | 2.21 (0.62-7.82) |  |
|  | Model 2 | 1.0 | - | - | 0.187 |  | 2.92 (0.61-13.98) |  |
| Gender |  |  |  |  |  |  |  |  |
| Male | Crude model | 1.0 | 1.33 (0.48-3.68) | **3.80 (1.57-9.21)** | **0.001** |  | 1.15 (0.93-1.42) | **0.006** |
|  | Model 1 | 1.0 | 1.25 (0.45-3.52) | **4.28 (1.73-10.62)** | **<0.001** |  | **1.66 (1.21-2.28)** | **0.017** |
|  | Model 2 | 1.0 | 1.31 (0.46-3.68) | **5.06 (1.99-12.90)** | **<0.001** |  | **1.84 (1.28-2.64)** | **0.019** |
| Female | Crude model | 1.0 | 0.29 (0.06-1.50) | 0.40 (0.09-1.67) | 0.299 |  | 1.15 (0.76-1.75) |  |
|  | Model 1 | 1.0 | 0.30 (0.06-1.54) | 0.49 (0.11-2.20) | 0.450 |  | 0.96 (0.51-1.82) |  |
|  | Model 2 | 1.0 | 0.31 (0.06-1.68) | 0.58 (0.11-3.01) | 0.616 |  | 1.12 (0.49-2.56) |  |
| Area |  |  |  |  |  |  |  |  |
| Qingdao | Crude model | 1.0 | 0.67 (0.24-1.86) | 2.23 (0.91-5.49) | **0.030** |  | 1.16 (0.62-2.16) | 0.578 |
|  | Model 1 | 1.0 | 0.61 (0.22-1.72) | 2.04 (0.82-5.10) | **0.046** |  | 1.10 (0.58-2.08) | 0.489 |
|  | Model 2 | 1.0 | 0.62 (0.22-1.77) | 2.49 (0.96-6.48) | **0.021** |  | 1.32 (0.64-2.71) | 0.340 |
| Linyi | Crude model | 1.0 | 1.34 (0.30-6.13) | 3.34 (0.91-12.25) | **0.034** |  | **1.45 (1.10-1.90)** |  |
|  | Model 1 | 1.0 | 1.34 (0.29-6.10) | 3.32 (0.90-12.27) | **0.037** |  | **1.45 (1.10-1.91)** |  |
|  | Model 2 | 1.0 | 1.54 (0.33-7.17) | **4.14 (1.04-16.45)** | **0.024** |  | **1.62 (1.14-2.31)** |  |
| BMI (kg/m^2^) |  |  |  |  |  |  |  |  |
| <24 | Crude model | 1.0 | 1.08 (0.41-2.86) | **2.65 (1.13-6.19)** | **0.008** |  | 1.20 (0.93-1.56) | 0.277 |
|  | Model 1 | 1.0 | 1.02 (0.38-2.71) | **2.84 (1.20-6.68)** | **0.002** |  | **1.40 (1.06-1.84)** | 0.354 |
|  | Model 2 | 1.0 | 1.04 (0.39-2.83) | **3.75 (1.51-9.31)** | **0.001** |  | **1.63 (1.15-2.31)** | 0.401 |
| ≥24 | Crude model | 1.0 | 0.49 (0.09-2.75) | 1.17 (0.30-4.52) | 0.666 |  | 1.28 (0.71-2.30) |  |
|  | Model 1 | 1.0 | 0.47 (0.08-2.69) | 1.11 (0.28-4.43) | 0.563 |  | 1.52 (0.75-3.09) |  |
|  | Model 2 | 1.0 | 0.46 (0.08-2.67) | 2.09 (0.44-9.87) | 0.285 |  | 1.93 (0.88-4.25) |  |
| Diabetes |  |  |  |  |  |  |  |  |
| No | Crude model | 1.0 | 0.96 (0.35-2.61) | **2.39 (1.02-5.59)** | **0.014** |  | 1.26 (0.99-1.61) | 0.801 |
|  | Model 1 | 1.0 | 0.88 (0.32-2.45) | **2.62 (1.10-6.22)** | **0.007** |  | **1.40 (1.08-1.81)** | 0.730 |
|  | Model 2 | 1.0 | 0.96 (0.34-2.66) | **3.29 (1.34-8.11)** | **0.002** |  | **1.65 (1.19-2.30)** | 0.591 |
| Yes | Crude model | 1.0 | 0.71 (0.16-3.21) | 2.24 (0.57-8.75) | 0.191 |  | 1.46 (0.45-4.72) |  |
|  | Model 1 | 1.0 | 0.62 (0.13-3.04) | 2.80 (0.64-12.31) | 0.128 |  | 1.44 (0.43-4.75) |  |
|  | Model 2 | 1.0 | 0.67 (0.14-3.28) | 3.02 (0.66-13.92) | 0.116 |  | 1.56 (0.43-5.64) |  |
| Smoking |  |  |  |  |  |  |  |  |
| No | Crude model | 1.0 | 0.55 (0.21-1.42) | 1.47 (0.70-3.09) | 0.121 |  | 1.16 (0.86-1.55) | 0.053 |
|  | Model 1 | 1.0 | 0.53 (0.20-1.38) | 1.89 (0.87-4.09) | **0.029** |  | **1.46 (1.06-2.02)** | 0.078 |
|  | Model 2 | 1.0 | 0.55 (0.21-1.45) | 2.14 (0.96-4.78) | **0.019** |  | **1.62 (1.12-2.35)** | 0.056 |
| Yes | Crude model | 1.0 | - | - | **0.014** |  | 1.33 (0.89-1.99) |  |
|  | Model 1 | 1.0 | - | - | **0.017** |  | 1.31 (0.76-2.26) |  |
|  | Model 2 | 1.0 | - | - | **0.008** |  | 2.07 (0.94-4.56) |  |
| **“Fruit, legumes, and eggs”** |  |  |  |  |  |  |  |  |
| Age (years) |  |  |  |  |  |  |  |  |
| ≤65 | Crude model | 1.0 | 0.97 (0.46-2.06) | 1.02 (0.48-2.13) | 0.951 |  | 0.95 (0.69-1.31) | 0.906 |
|  | Model 1 | 1.0 | 0.69 (0.32-1.52) | 0.72 (0.33-1.57) | 0.512 |  | 0.87 (0.58-1.29) | 0.864 |
|  | Model 2 | 1.0 | 0.71 (0.32-1.59) | 0.81 (0.33-1.96) | 0.768 |  | 0.93 (0.55-1.58) | 0.906 |
| >65 | Crude model | 1.0 | 1.12 (0.07-18.52) | 1.21 (0.07-20.01) | 0.897 |  | 1.03 (0.27-3.99) |  |
|  | Model 1 | 1.0 | 0.76 (0.04-13.87) | 0.69 (0.04-13.10) | 0.820 |  | 0.77 (0.15-4.01) |  |
|  | Model 2 | 1.0 | 0.85 (0.04-19.95) | 0.84 (0.01-50.17) | 0.944 |  | 0.80 (0.08-7.60) |  |
| Gender |  |  |  |  |  |  |  |  |
| Male | Crude model | 1.0 | 0.77 (0.33-1.79) | 0.94 (0.43-2.08) | 0.976 |  | 0.94 (0.67-1.32) | 0.624 |
|  | Model 1 | 1.0 | 0.50 (0.21-1.22) | 0.62 (0.27-1.45) | 0.452 |  | 0.85 (0.54-1.33) | 0.619 |
|  | Model 2 | 1.0 | 0.48 (0.19-1.20) | 0.61 (0.23-1.63) | 0.511 |  | 0.82 (0.46-1.46) | 0.689 |
| Female | Crude model | 1.0 | 2.11 (0.48-9.26) | 1.44 (0.28-7.48) | 0.742 |  | 1.13 (0.47-2.69) |  |
|  | Model 1 | 1.0 | 1.97 (0.43-8.95) | 1.28 (0.24-6.90) | 0.879 |  | 1.06 (0.42-2.64) |  |
|  | Model 2 | 1.0 | 2.25 (0.46-11.11) | 1.84 (0.27-12.57) | 0.621 |  | 1.55 (0.41-5.85) |  |
| Area |  |  |  |  |  |  |  |  |
| Qingdao | Crude model | 1.0 | 0.70 (0.26-1.87) | 0.61 (0.22-1.64) | 0.383 |  | 0.79 (0.45-1.41) | 0.525 |
|  | Model 1 | 1.0 | 0.67 (0.25-1.83) | 0.59 (0.21-1.62) | 0.375 |  | 0.78 (0.43-1.41) | 0.541 |
|  | Model 2 | 1.0 | 0.67 (0.24-1.86) | 0.67 (0.21-2.10) | 0.598 |  | 0.89 (0.44-1.79) | 0.491 |
| Linyi | Crude model | 1.0 | 0.67 (0.20-2.28) | 1.04 (0.35-3.09) | 0.895 |  | 0.96 (0.61-1.53) |  |
|  | Model 1 | 1.0 | 0.68 (0.20-2.31) | 1.07 (0.36-3.18) | 0.864 |  | 0.97 (0.61-1.54) |  |
|  | Model 2 | 1.0 | 0.68 (0.20-2.40) | 1.05 (0.29-3.88) | 0.902 |  | 0.93 (0.46-1.89) |  |
| BMI (kg/m^2^) |  |  |  |  |  |  |  |  |
| <24 | Crude model | 1.0 | 1.19 (0.53-2.70) | 1.12 (0.49-2.58) | 0.823 |  | 0.98 (0.70-1.37) | 0.476 |
|  | Model 1 | 1.0 | 1.15 (0.51-2.61) | 1.09 (0.47-2.51) | 0.594 |  | 0.87 (0.56-1.37) | 0.554 |
|  | Model 2 | 1.0 | 0.86 (0.36-2.06) | 0.79 (0.29-2.15) | 0.667 |  | 0.86 (0.49-1.54) | 0.572 |
| ≥24 | Crude model | 1.0 | 0.36 (0.07-1.84) | 0.55 (0.13-2.35) | 0.643 |  | 0.85 (0.35-2.08) |  |
|  | Model 1 | 1.0 | 0.34 (0.06-1.76) | 0.51 (0.12-2.25) | 0.572 |  | 0.81 (0.32-2.06) |  |
|  | Model 2 | 1.0 | 0.31 (0.05-1.86) | 0.65 (0.10-4.02) | 0.976 |  | 1.17 (0.35-2.90) |  |
| Diabetes |  |  |  |  |  |  |  |  |
| No | Crude model | 1.0 | 0.94 (0.41-2.17) | 1.15 (0.51-2.60) | 0.710 |  | 0.95 (0.67-1.36) | 0.243 |
|  | Model 1 | 1.0 | 0.74 (0.31-1.74) | 0.98 (0.43-2.27) | 0.935 |  | 0.91 (0.60-1.39) | 0.249 |
|  | Model 2 | 1.0 | 0.74 (0.30-1.78) | 1.12 (0.43-2.93) | 0.718 |  | 0.96 (0.54-1.70) | 0.262 |
| Yes | Crude model | 1.0 | 0.61 (0.13-3.00) | 0.40 (0.08-1.93) | 0.267 |  | 0.86 (0.38-1.97) |  |
|  | Model 1 | 1.0 | 0.42 (0.08-2.35) | 0.25 (0.04-1.37) | 0.141 |  | 0.72 (0.28-1.82) |  |
|  | Model 2 | 1.0 | 0.42 (0.07-2.44) | 0.23 (0.03-1.58) | 0.165 |  | 0.75 (0.25-2.25) |  |
| Smoking |  |  |  |  |  |  |  |  |
| No | Crude model | 1.0 | 1.01 (0.45-2.28) | 1.27 (0.58-2.80) | 0.525 |  | 1.09 (0.69-1.72) | 0.356 |
|  | Model 1 | 1.0 | 0.63 (0.27-1.50) | 0.77 (0.33-1.78) | 0.708 |  | 0.83 (0.49-1.41) | 0.696 |
|  | Model 2 | 1.0 | 0.61 (0.25-1.49) | 0.81 (0.31-2.13) | 0.862 |  | 0.82 (0.43-1.56) | 0.752 |
| Yes | Crude model | 1.0 | 0.93 (0.19-4.50) | 0.55 (0.10-2.90) | 0.439 |  | 0.88 (0.52-1.49) |  |
|  | Model 1 | 1.0 | 1.06 (0.20-5.56) | 0.53 (0.09-2.97) | 0.403 |  | 0.85 (0.47-1.53) |  |
|  | Model 2 | 1.0 | 1.13 (0.21-6.13) | 0.66 (0.10-4.42) | 0.609 |  | 1.09 (0.47-2.54) |  |

a. The lowest quantile was used as the reference group. Statistically significant values (P<0.05) are shown in bold characters.

b. Model 1 was adjusted for age, gender, and area. Model 2 was additionally adjusted for BMI, energy intake, and diabetes status.

c. The value was not calculated when the number of patients attributed to the reference group was 0 in the multivariate logistic regressions.

# Table S3. Multivariate odds ratio of liver dysfunction stratified by age, gender, area, BMI, diabetes, smoking.

|  | | **Multivariate relative risk (95% CI) per tertiles^a^** | | | ***P* values for trend** |  | **OR per SD** | ***P* values for interaction** |
| --- | --- | --- | --- | --- | --- | --- | --- | --- |
|  |  | **Tertile 1 (lowest)** | **Tertile 2** | **Tertile 3 (highest)** |  |  |  |  |
| **China healthy diet index** |  |  |  |  |  |  |  |  |
| Age (years) |  |  |  |  |  |  |  |  |
| ≤65 | Crude model | 1.0 | 0.83 (0.49-1.38) | 1.12 (0.69-1.84) | 0.499 |  | 1.04 (0.84-1.29) | 0.339 |
|  | Model 1 | 1.0 | **0.53 (0.30-0.95)** | **0.20 (0.09-0.44)** | **<0.001** |  | **0.49 (0.35-0.68)** | 0.345 |
|  | Model 2 | 1.0 | **0.52 (0.29-0.93)** | **0.18 (0.08-0.40)** | **<0.001** |  | **0.44 (0.32-0.62)** | 0.375 |
| >65 | Crude model | 1.0 | 2.97 (0.70-12.68) | 2.48 (0.52-11.85) | 0.295 |  | 1.36 (0.80-2.29) |  |
|  | Model 1 | 1.0 | 1.59 (0.30-8.37) | 0.09 (0.01-1.82) | 0.100 |  | 0.60 (0.27-1.31) |  |
|  | Model 2 | 1.0 | 1.32 (0.22-7.89) | 0.06 (0.00-1.62) | 0.156 |  | 0.48 (0.19-1.22) |  |
| Gender |  |  |  |  |  |  |  |  |
| Male | Crude model | 1.0 | 1.19 (0.69-2.06) | 1.63 (0.96-2.77) | 0.065 |  | 1.15 (0.93-1.42) | 0.178 |
|  | Model 1 | 1.0 | 0.60 (0.31-1.15) | **0.21 (0.09-0.50)** | **<0.001** |  | **0.49 (0.35-0.68)** | 0.450 |
|  | Model 2 | 1.0 | 0.60 (0.31-1.16) | **0.20 (0.08-0.51)** | **0.001** |  | **0.46 (0.32-0.65)** | 0.222 |
| Female | Crude model | 1.0 | 0.67 (0.26-1.75) | 0.78 (0.30-1.99) | 0.662 |  | 1.13 (0.74-1.71) |  |
|  | Model 1 | 1.0 | 0.67 (0.24-1.84) | **0.16 (0.04-0.68)** | **0.013** |  | 0.65 (0.37-1.15) |  |
|  | Model 2 | 1.0 | 0.43 (0.13-1.37) | **0.07 (0.01-0.36)** | **0.001** |  | **0.45 (0.23-0.85)** |  |
| Area |  |  |  |  |  |  |  |  |
| Qingdao | Crude model | 1.0 | 0.18 (0.02-1.62) | **0.06 (0.01-0.46)** | **<0.001** |  | **0.32 (0.21-0.50)** | **0.025** |
|  | Model 1 | 1.0 | 0.13 (0.01-1.22) | **0.04 (0.01-0.38)** | **<0.001** |  | **0.33 (0.21-0.52)** | **0.030** |
|  | Model 2 | 1.0 | 0.15 (0.02-1.42) | **0.05 (0.01-0.40)** | **<0.001** |  | **0.31 (0.19-0.51)** | **0.027** |
| Linyi | Crude model | 1.0 | 0.70 (0.39-1.24) | 0.40 (0.12-1.37) | 0.080 |  | 0.83 (0.56-1.22) |  |
|  | Model 1 | 1.0 | 0.68 (0.38-1.22) | 0.39 (0.11-1.34) | 0.073 |  | 0.81 (0.54-1.21) |  |
|  | Model 2 | 1.0 | 0.59 (0.32-1.08) | 0.33 (0.09-1.19) | **0.035** |  | 0.71 (0.46-1.08) |  |
| BMI (kg/m^2^) |  |  |  |  |  |  |  |  |
| <24 | Crude mode | 1.0 | 1.12 (0.67-1.87) | 1.32 (0.79-2.21) | **0.287** |  | 1.13 (0.91-1.39) | 0.888 |
|  | Model 1 | 1.0 | 0.69 (0.38-1.22) | **0.16 (0.07-0.38)** | **<0.001** |  | **0.49 (0.36-0.68)** | 0.890 |
|  | Model 2 | 1.0 | 0.64 (0.35-1.15) | **0.15 (0.06-0.37)** | **<0.001** |  | **0.46 (0.33-0.64)** | 0.995 |
| ≥24 | Crude model | 1.0 | 0.56 (0.15-2.13) | 1.07 (0.36-3.23) | 0.640 |  | 1.06 (0.65-1.71) |  |
|  | Model 1 | 1.0 | 0.35 (0.07-1.65) | 0.32 (0.06-1.88) | 0.296 |  | 0.69 (0.34-1.38) |  |
|  | Model 2 | 1.0 | 0.28 (0.05-1.61) | 0.26 (0.04-1.80) | 0.279 |  | 0.65 (0.31-1.38) |  |
| Diabetes |  |  |  |  |  |  |  |  |
| No | Crude model | 1.0 | 1.02 (0.63-1.66) | 1.18 (0.69-2.03) | 0.543 |  | 1.13 (0.90-1.42) | 0.777 |
|  | Model 1 | 1.0 | 0.65 (0.37-1.13) | **0.17 (0.07-0.39)** | **<0.001** |  | **0.55 (0.40-0.76)** | 0.498 |
|  | Model 2 | 1.0 | 0.61 (0.34-1.08) | **0.13 (0.05-0.31)** | **<0.001** |  | **0.48 (0.34-0.67)** | 0.497 |
| Yes | Crude model | 1.0 | 1.00 (0.13-7.89) | 1.36 (0.26-7.23) | 0.599 |  | 0.85 (0.52-1.38) |  |
|  | Model 1 | 1.0 | 0.29 (0.02-4.73) | 0.24 (0.02-3.24) | 0.387 |  | **0.47 (0.24-0.95)** |  |
|  | Model 2 | 1.0 | 0.29 (0.02-5.59) | 0.26 (0.02-4.24) | 0.506 |  | 0.47 (0.22-1.00) |  |
| Smoking |  |  |  |  |  |  |  |  |
| No | Crude model | 1.0 | 0.81 (0.47-1.41) | 1.17 (0.70-1.95) | 0.365 |  | 1.07 (0.87-1.32) | 0.582 |
|  | Model 1 | 1.0 | **0.42 (0.22-0.82)** | **0.14 (0.06-0.33)** | **<0.001** |  | **0.45 (0.33-0.62)** | 0.139 |
|  | Model 2 | 1.0 | **0.40 (0.20-0.80)** | **0.13 (0.05-0.31)** | **<0.001** |  | **0.41 (0.29-0.58)** | 0.132 |
| Yes | Crude model | 1.0 | 1.88 (0.73-4.80) | 1.36 (0.33-5.64) | 0.454 |  | 1.41 (0.84-2.37) |  |
|  | Model 1 | 1.0 | 1.48 (0.55-4.00) | 0.47 (0.06-3.55) | 0.713 |  | 1.08 (0.54-2.17) |  |
|  | Model 2 | 1.0 | 1.35 (0.48-3.86) | 0.26 (0.03-2.55) | 0.443 |  | 0.95 (0.44-2.05) |  |
| **“Vegetables, red meat, fish, and other seafood”** |  |  |  |  |  |  |  |  |
| Age (years) |  |  |  |  |  |  |  |  |
| ≤65 | Crude model | 1.0 | 1.59 (0.93-2.73) | **2.07 (1.24-3.46)** | **0.008** |  | 1.19 (0.98-1.46) | 0.191 |
|  | Model 1^b^ | 1.0 | 1.18 (0.66-2.11) | 0.69 (0.34-1.41) | 0.180 |  | **0.71 (0.54-0.94)** | 0.194 |
|  | Model 2 | 1.0 | 1.37 (0.74-2.55) | 0.78 (0.38-1.61) | 0.250 |  | 0.76 (0.57-1.00) | 0.224 |
| >65 | Crude model | 1.0 | 2.85 (0.53-15.44) | **6.39 (1.20-34.13)** | **0.023** |  | **1.91 (1.13-3.22)** |  |
|  | Model 1 | 1.0 | 2.43 (0.41-14.32) | 2.37 (0.25-22.66) | 0.598 |  | 1.37 (0.65-2.88) |  |
|  | Model 2 | 1.0 | 3.35 (0.53-21.17) | 2.42 (0.23-25.26) | 0.645 |  | 1.20 (0.54-2.68) |  |
| Gender |  |  |  |  |  |  |  |  |
| Male | Crude model | 1.0 | **1.81 (1.02-3.24)** | **2.46 (1.43-4.24)** | **0.002** |  | **1.30 (1.06-1.59)** | 0.896 |
|  | Model 1 | 1.0 | 1.20 (0.64-2.5) | 0.63 (0.29-1.38) | 0.137 |  | **0.74 (0.56-0.98)** | 0.900 |
|  | Model 2 | 1.0 | 1.43 (0.73-2.80) | 0.71 (0.32-1.58) | 0.190 |  | 0.79 (0.59-1.04) | 0.859 |
| Female | Crude model | 1.0 | 1.61 (0.57-4.57) | 2.61 (0.89-7.67) | 0.079 |  | 1.32 (0.85-2.06) |  |
|  | Model 1 | 1.0 | 1.34 (0.45-3.98) | 1.36 (0.33-5.58) | 0.747 |  | 0.87 (0.46-1.63) |  |
|  | Model 2 | 1.0 | 1.76 (0.54-5.73) | 1.52 (0.35-6.62) | 0.744 |  | 0.87 (0.47-1.63) |  |
| Area |  |  |  |  |  |  |  |  |
| Qingdao | Crude model | 1.0 | 1.00 (0.18-5.51) | 0.54 (0.11-2.76) | 0.063 |  | **0.69 (0.49-0.96)** | 0.084 |
|  | Model 1 | 1.0 | 1.03 (0.18-5.78) | 0.51 (0.10-2.68) | **0.043** |  | **0.68 (0.48-0.96)** | 0.077 |
|  | Model 2 | 1.0 | 0.81 (0.14-4.76) | 0.47 (0.09-2.65) | 0.129 |  | 0.70 (0.45-1.09) | **0.023** |
| Linyi | Crude model | 1.0 | 1.14 (0.63-2.04) | 1.20 (0.49-2.97) | 0.650 |  | 0.91 (0.60-1.39) |  |
|  | Model 1 | 1.0 | 1.15 (0.64-2.08) | 1.16 (0.47-2.89) | 0.693 |  | 0.91 (0.60-1.39) |  |
|  | Model 2 | 1.0 | 1.59 (0.84-3.01) | 1.47 (0.58-3.74) | 0.327 |  | 1.17 (0.77-1.77) |  |
| BMI (kg/m^2^) |  |  |  |  |  |  |  |  |
| <24 | Crude mode | 1.0 | **1.80 (1.05-3.09)** | **2.48 (1.45-4.24)** | **0.002** |  | **1.31 (1.07-1.62)** | 0.835 |
|  | Model 1 | 1.0 | 1.31 (0.73-2.34) | 0.74 (0.36-1.55) | 0.239 |  | 0.78 (0.59-1.03) | 0.662 |
|  | Model 2 | 1.0 | 1.65 (0.88-3.09) | 0.87 (0.41-1.84) | 0.355 |  | 0.82 (0.62-1.07) | 0.839 |
| ≥24 | Crude model | 1.0 | 1.07 (0.27-4.18) | 1.97 (0.60-6.42) | 0.180 |  | 1.14 (0.74-1.78) |  |
|  | Model 1 | 1.0 | 0.89 (0.20-4.01) | 0.94 (0.14-6.37) | 0.989 |  | 0.71 (0.36-1.39) |  |
|  | Model 2 | 1.0 | 0.87 (0.18-4.14) | 0.94 (0.14-6.39) | 0.992 |  | 0.70 (0.36-1.39) |  |
| Diabetes |  |  |  |  |  |  |  |  |
| No | Crude model | 1.0 | 1.65 (0.98-2.79) | **2.54 (1.47-4.37)** | **0.001** |  | **1.29 (1.04-1.61)** | 0.497 |
|  | Model 1 | 1.0 | 1.22 (0.69-2.14) | 0.80 (0.38-1.68) | 0.416 |  | 0.75 (0.56-1.00) | 0.848 |
|  | Model 2 | 1.0 | 1.53 (0.84-2.80) | 0.92 (0.43-1.97) | 0.542 |  | 0.80 (0.60-1.06) | 0.612 |
| Yes | Crude model | 1.0 | 1.82 (0.28-11.87) | 1.80 (0.36-9.17) | 0.604 |  | 1.16 (0.71-1.88) |  |
|  | Model 1 | 1.0 | 1.15 (0.15-8.99) | 0.68 (0.10-4.61) | 0.440 |  | 0.86 (0.48-1.51) |  |
|  | Model 2 | 1.0 | 1.03 (0.12-9.00) | 0.58 (0.08-4.44) | 0.434 |  | 0.83 (0.39-1.79) |  |
| Smoking |  |  |  |  |  |  |  |  |
| No | Crude model | 1.0 | 1.74 (0.95-3.20) | **2.67 (1.50-4.74)** | **0.001** |  | **1.33 (1.08-1.65)** | 0.597 |
|  | Model 1 | 1.0 | 1.20 (0.63-2.32) | 0.73 (0.34-1.59) | 0.215 |  | **0.72 (0.53-0.99)** | 0.778 |
|  | Model 2 | 1.0 | 1.30 (0.65-2.58) | 0.80 (0.37-1.76) | 0.298 |  | 0.75 (0.55-1.02) | 0.716 |
| Yes | Crude model | 1.0 | 1.62 (0.63-4.14) | 1.55 (0.37-6.48) | 0.435 |  | 1.11 (0.71-1.75) |  |
|  | Model 1 | 1.0 | 1.27 (0.46-3.53) | 0.80 (0.14-4.65) | 0.869 |  | 0.89 (0.54-1.49) |  |
|  | Model 2 | 1.0 | 2.51 (0.77-8.13) | 0.84 (0.14-5.02) | 0.972 |  | 1.05 (0.63-1.73) |  |
| **“Organ meat, poultry, vegetable oil”** |  |  |  |  |  |  |  |  |
| Age (years) |  |  |  |  |  |  |  |  |
| ≤65 | Crude mode | 1.0 | 1.28 (0.77-2.13) | 1.59 (0.96-2.63) | 0.083 |  | 1.19 (0.98-1.44) | 0.408 |
|  | Model 1 | 1.0 | 1.31 (0.78-2.21) | **1.89 (1.12-3.21)** | **0.018** |  | **1.34 (1.09-1.66)** | 0.440 |
|  | Model 2 | 1.0 | 1.34 (0.79-2.27) | **1.76 (1.02-3.06)** | 0.050 |  | **1.29 (1.03-1.61)** | 0.448 |
| >65 | Crude model | 1.0 | 0.69 (0.11-4.34) | 2.53 (0.63-10.27) | 0.090 |  | 1.13 (0.67-1.91) |  |
|  | Model 1 | 1.0 | 0.73 (0.11-4.97) | 3.58 (0.79-16.34) | **0.041** |  | 1.52 (0.81-2.85) |  |
|  | Model 2 | 1.0 | 0.73 (0.10-5.12) | 3.44 (0.69-17.13) | 0.069 |  | 1.37 (0.66-2.85) |  |
| Gender |  |  |  |  |  |  |  |  |
| Male | Crude model | 1.0 | 1.59 (0.91-2.77) | **2.15 (1.25-3.72)** | **0.009** |  | 1.23 (0.98-1.53) | 0.054 |
|  | Model 1 | 1.0 | 1.56 (0.87-2.80) | **2.43 (1.37-4.32)** | **0.003** |  | **1.39 (1.09-1.77)** | 0.191 |
|  | Model 2 | 1.0 | 1.61 (0.90-2.91) | **2.28 (1.25-4.14)** | **0.010** |  | **1.32 (1.03-1.70)** | 0.218 |
| Female | Crude model | 1.0 | 0.57 (0.21-1.52) | 0.73 (0.29-1.83) | 0.685 |  | 1.10 (0.81-1.49) |  |
|  | Model 1 | 1.0 | 0.57 (0.21-1.62) | 1.04 (0.39-2.79) | 0.735 |  | 1.27 (0.91-1.76) |  |
|  | Model 2 | 1.0 | 0.56 (0.20-1.57) | 0.70 (0.23-2.16) | 0.670 |  | 1.03 (0.65-1.64) |  |
| Area |  |  |  |  |  |  |  |  |
| Qingdao | Crude model | 1.0 | 0.75 (0.39-1.46) | 1.36 (0.68-2.73) | 0.272 |  | 1.13 (0.70-1.81) | 0.106 |
|  | Model 1 | 1.0 | 0.71 (0.36-1.38) | 1.25 (0.61-2.54) | 0.382 |  | 1.08 (0.67-1.75) | 0.060 |
|  | Model 2 | 1.0 | 0.69 (0.35-1.38) | 1.28 (0.61-2.67) | 0.366 |  | 1.11 (0.67-1.85) | 0.079 |
| Linyi | Crude model | 1.0 | **2.60 (1.14-5.94)** | **3.36 (1.53-7.40)** | **0.006** |  | **1.35 (1.10-1.67)** |  |
|  | Model 1 | 1.0 | **2.60 (1.14-5.94)** | **3.53 (1.59-7.81)** | **0.004** |  | **1.40 (1.12-1.74)** |  |
|  | Model 2 | 1.0 | **2.58 (1.12-5.96)** | **2.88 (1.25-6.61)** | **0.037** |  | **1.28 (1.01-1.61)** |  |
| BMI (kg/m^2^) |  |  |  |  |  |  |  |  |
| <24 | Crude mode | 1.0 | 1.28 (0.74-2.19) | **1.87 (1.11-3.15)** | **0.016** |  | **1.20 (1.00-1.45)** | 0.221 |
|  | Model 1 | 1.0 | 1.26 (0.72-2.22) | **2.25 (1.30-3.90)** | **0.002** |  | **1.39 (1.13-1.71)** | 0.303 |
|  | Model 2 | 1.0 | 1.23 (0.70-2.17) | **1.95 (1.11-3.45)** | **0.017** |  | **1.29 (1.03-1.61)** | 0.270 |
| ≥24 | Crude model | 1.0 | 1.29 (0.43-3.90) | **0.88 (0.29-2.64)** | 0.724 |  | 0.89 (0.54-1.45) |  |
|  | Model 1 | 1.0 | 1.22 (0.39-3.80) | 1.02 (0.32-3.30) | 0.979 |  | 1.05 (0.60-1.84) |  |
|  | Model 2 | 1.0 | 1.22 (0.39-3.82) | 1.02 (0.29-3.60) | 0.991 |  | 1.06 (0.59-1.91) |  |
| Diabetes |  |  |  |  |  |  |  |  |
| No | Crude model | 1.0 | 1.59 (0.91-2.79) | **1.98 (1.15-3.40)** | **0.022** |  | **1.20 (1.00-1.44)** | 0.323 |
|  | Model 1 | 1.0 | 1.54 (0.86-2.77) | **2.25 (1.28-3.98)** | **0.006** |  | **1.36 (1.11-1.66)** | 0.243 |
|  | Model 2 | 1.0 | 1.57 (0.87-2.84) | **2.09 (1.16-3.78)** | **0.021** |  | **1.30 (1.05-1.61)** | 0.275 |
| Yes | Crude model | 1.0 | 0.61 (0.22-1.69) | 1.18 (0.40-3.48) | 0.688 |  | 0.88 (0.36-2.17) |  |
|  | Model 1 | 1.0 | 0.58 (0.20-1.64) | 1.27 (0.41-3.95) | 0.603 |  | 0.86 (0.34-2.16) |  |
|  | Model 2 | 1.0 | 0.58 (0.20-1.68) | 1.20 (0.38-3.82) | 0.693 |  | 0.77 (0.30-1.97) |  |
| Smoking |  |  |  |  |  |  |  |  |
| No | Crude model | 1.0 | 0.93 (0.55-1.58) | 1.29 (0.78-2.12) | 0.237 |  | 1.00 (0.81-1.24) | **0.049** |
|  | Model 1 | 1.0 | 0.92 (0.53-1.61) | 1.68 (0.98-2.87) | **0.030** |  | 1.24 (0.98-1.56) | 0.116 |
|  | Model 2 | 1.0 | 0.94 (0.53-1.65) | 1.62 (0.91-2.86) | 0.064 |  | 1.19 (0.92-1.54) | 0.093 |
| Yes | Crude model | 1.0 | **6.84 (1.45-32.35)** | **7.69 (1.56-37.97)** | **0.033** |  | **2.09 (1.16-3.78)** |  |
|  | Model 1 | 1.0 | **5.89 (1.22-28.40)** | **7.15 (1.41-36.29)** | **0.043** |  | **2.30 (1.20-4.40)** |  |
|  | Model 2 | 1.0 | **7.00 (1.39-35.23)** | **6.75 (1.28-35.52)** | 0.083 |  | **2.11 (1.10-4.04)** |  |
| **“Fruit, legumes, and eggs”** |  |  |  |  |  |  |  |  |
| Age (years) |  |  |  |  |  |  |  |  |
| ≤65 | Crude mode | 1.0 | 1.41 (0.86-2.32) | 1.00 (0.60-1.67) | 0.775 |  | 1.06 (0.88-1.27) | **0.028** |
|  | Model 1 | 1.0 | 1.05 (0.62-1.77) | 0.72 (0.42-1.24) | 0.166 |  | 1.01 (0.82-1.2) | 0.018 |
|  | Model 2 | 1.0 | 0.92 (0.53-1.58) | 0.54 (0.29-1.01) | **0.036** |  | 0.82 (0.60-1.11) | **0.023** |
| >65 | Crude model | 1.0 | 1.73 (0.28-10.90) | **5.81 (1.15-29.21)** | **0.016** |  | 1.64 (0.94-2.86) |  |
|  | Model 1 | 1.0 | 1.50 (0.23-9.93) | 4.87 (0.92-25.89) | **0.033** |  | 1.54 (0.86-2.76) |  |
|  | Model 2 | 1.0 | 1.63 (0.23-11.75) | 7.24 (0.90-58.37) | **0.039** |  | 1.46 (0.72-2.99) |  |
| Gender |  |  |  |  |  |  |  |  |
| Male | Crude model | 1.0 | 1.54 (0.90-2.65) | 1.20 (0.69-2.08) | 0.749 |  | 1.04 (0.86-1.26) | 0.785 |
|  | Model 1 | 1.0 | 1.13 (0.64-2.01) | 0.87 (0.48-1.56) | 0.495 |  | **1.39 (1.09-1.77)** | 0.795 |
|  | Model 2 | 1.0 | 1.00 (0.55-1.83) | 0.67 (0.34-1.32) | 0.181 |  | 0.84 (0.61-1.15) | 0.753 |
| Female | Crude model | 1.0 | 1.12 (0.43-2.87) | 1.36 (0.53-3.54) | 0.521 |  | 1.73 (1.00-2.97) |  |
|  | Model 1 | 1.0 | 0.96 (0.36-2.56) | 1.12 (0.41-3.03) | 0.804 |  | 1.27 (0.91-1.76) |  |
|  | Model 2 | 1.0 | 0.75 (0.27-2.07) | 0.72 (0.23-2.19) | 0.598 |  | 1.29 (0.62-2.69) |  |
| Area |  |  |  |  |  |  |  |  |
| Qingdao | Crude model | 1.0 | 0.77 (0.36-1.62) | **0.46 (0.21-0.98)** | **0.030** |  | 0.68 (0.45-1.03) | **0.010** |
|  | Model 1 | 1.0 | 0.74 (0.35-1.59) | **0.44 (0.20-0.95)** | **0.026** |  | 0.67 (0.44-1.02) | **0.011** |
|  | Model 2 | 1.0 | 0.73 (0.33-1.61) | 0.49 (0.21-1.19) | 0.110 |  | 0.72 (0.43-1.19) | 0.063 |
| Linyi | Crude model | 1.0 | 1.27 (0.64-2.52) | 1.65 (0.85-3.19) | 0.138 |  | 1.21 (0.99-1.49) |  |
|  | Model 1 | 1.0 | 1.26 (0.64-2.51) | 1.64 (0.84-3.17) | 0.147 |  | 1.21 (0.99-1.48) |  |
|  | Model 2 | 1.0 | 1.05 (0.52-2.12) | 0.97 (0.44-2.15) | 0.922 |  | 0.92 (0.67-1.26) |  |
| BMI (kg/m^2^) |  |  |  |  |  |  |  |  |
| <24 | Crude mode | 1.0 | 1.51 (0.91-2.50) | 1.28 (0.76-2.15) | 0.482 |  | 1.12 (0.93-1.35) | 0.528 |
|  | Model 1 | 1.0 | 1.16 (0.68-1.99) | 0.93 (0.53-1.61) | 0.671 |  | 1.08 (0.88-1.32) | 0.643 |
|  | Model 2 | 1.0 | 1.02 (0.59-1.77) | 0.67 (0.35-1.27) | 0.166 |  | 0.84 (0.63-1.13) | 0.632 |
| ≥24 | Crude model | 1.0 | 0.91 (0.27-3.12) | 0.85 (0.25-2.83) | 0.792 |  | 0.99 (0.53-1.85) |  |
|  | Model 1 | 1.0 | 0.62 (0.16-2.33) | 0.63 (0.18-2.29) | 0.619 |  | 0.92 (0.48-1.79) |  |
|  | Model 2 | 1.0 | 0.60 (0.15-2.32) | 0.57 (0.13-2.53) | 0.580 |  | 0.87 (0.35-2.16) |  |
| Diabetes |  |  |  |  |  |  |  |  |
| No | Crude model | 1.0 | 1.47 (0.88-2.45) | 1.40 (0.83-2.37) | 0.268 |  | 1.12 (0.94-1.35) | 0.055 |
|  | Model 1 | 1.0 | 0.83 (0.48-1.44) | 0.97 (0.56-1.66) | 0.544 |  | 0.11 (0.92-1.35) | **0.045** |
|  | Model 2 | 1.0 | 1.17 (0.62-2.20) | 1.18 (0.66-2.11) | 0.605 |  | 0.89 (0.66-1.19) | 0.072 |
| Yes | Crude model | 1.0 | 0.70 (0.20-2.47) | 0.41 (0.12-1.42) | 0.124 |  | 0.88 (0.48-1.61) |  |
|  | Model 1 | 1.0 | 3.68 (0.94-14.49) | 1.79 (0.67-4.77) | 0.059 |  | 0.79 (0.42-1.50) |  |
|  | Model 2 | 1.0 | 5.26 (0.99-27.90) | 2.04 (0.70-5.97) | 0.054 |  | 0.78 (0.36-1.70) |  |
| Smoking |  |  |  |  |  |  |  |  |
| No | Crude model | 1.0 | 1.47 (0.88-2.44) | 1.34 (0.80-2.27) | 0.350 |  | 1.18 (0.88-1.58) | 0.739 |
|  | Model 1 | 1.0 | 0.97 (0.56-1.68) | 0.83 (0.47-1.46) | 0.483 |  | 0.91 (0.65-1.26) | 0.683 |
|  | Model 2 | 1.0 | 0.87 (0.49-1.56) | 0.73 (0.38-1.41) | 0.342 |  | 0.78 (0.51-1.18) | 0.908 |
| Yes | Crude model | 1.0 | 1.53 (0.47-5.02) | 1.18 (0.36-3.84) | 0.976 |  | 1.14 (0.90-1.43) |  |
|  | Model 1 | 1.0 | 1.63 (0.48-5.52) | 1.20 (0.36-3.96) | 0.991 |  | 1.15 (0.91-1.45) |  |
|  | Model 2 | 1.0 | 1.36 (0.38-4.78) | 0.62 (0.15-2.51) | 0.319 |  | 0.85 (0.54-1.32) |  |

a. The lowest quantile was used as the reference group. Statistically significant values (*P*<0.05) are shown in bold characters.

b. Model 1 was adjusted for age, gender, and area. Model 2 was additionally adjusted for BMI, energy intake, and diabetes status.

**
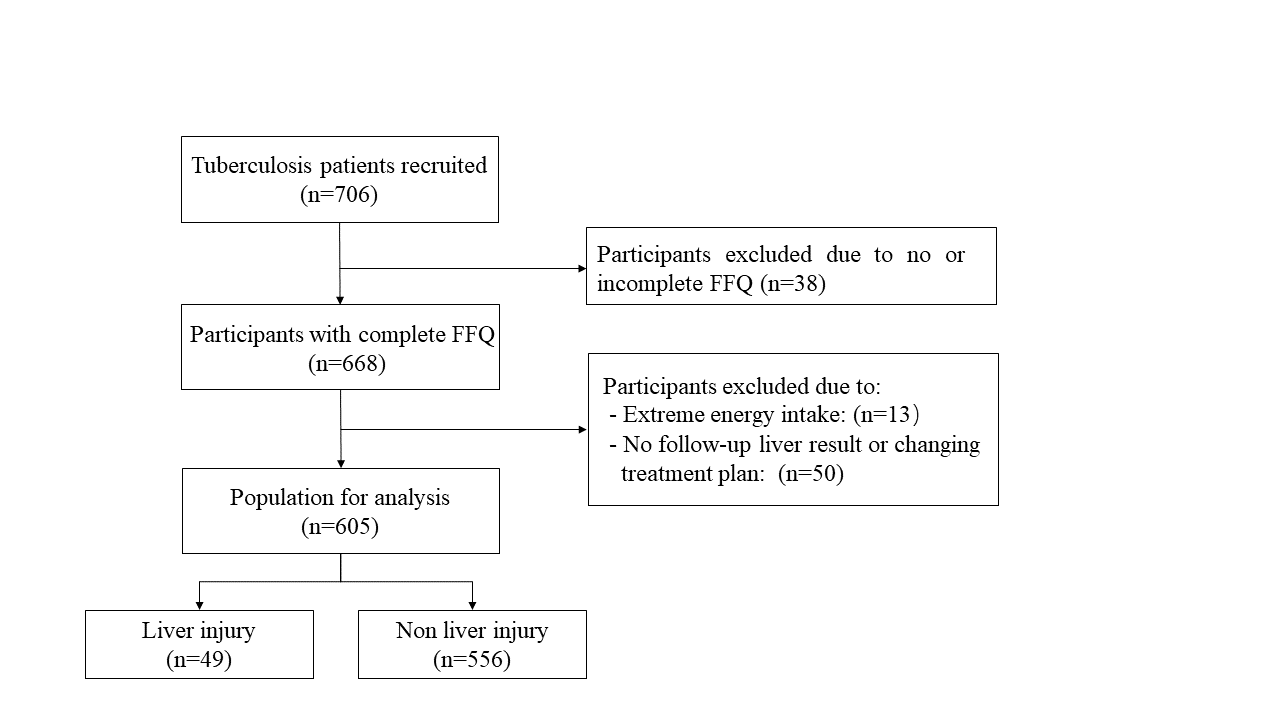
**

# Figure S1. The study flow chart.

# FFQ, food-frequency questionnaire.

**
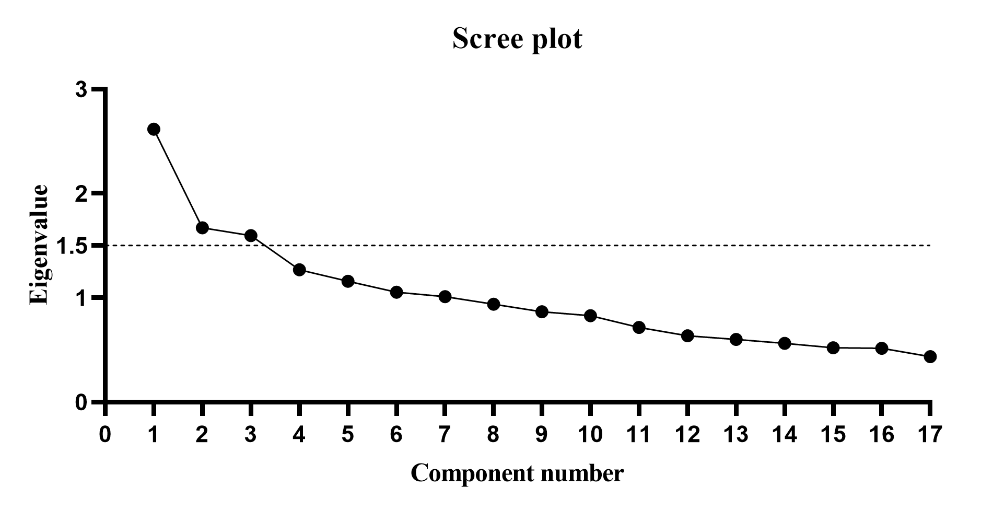
**

**Figure S2. Scree plot for dietary pattern identification by principal component analysis.**

The top three dietary patterns with eigenvalues >1.5 were extracted and used for subsequent analyses.
